# Could Empathy Be Taught? The Role of Advanced Technologies to Foster Empathy in Medical Students and Healthcare Professionals: A Systematic Review

**DOI:** 10.1007/s10916-025-02144-9

**Published:** 2025-01-14

**Authors:** Giorgio Li Pira, Chiara Ruini, Francesca Vescovelli, Rosa Baños, Sara Ventura

**Affiliations:** 1 Department of Psychology, University of Bologna, Bologna, Italy; 2 Department for Life Quality Studies, University of Bologna, Rimini, Italy; 3 Instituto Polibienestar, University of Valencia, Valencia, Spain; 4 CIBER of Physiopathology of Obesity and Nutrition (CIBEROBN), Madrid, Spain

**Keywords:** Empathy, Empathy-training, Healthcare worker, Digital technologies

## Abstract

The physician-patient relationship relies mostly on doctors’ empathetic abilities to understand and manage patients’ emotions, enhancing patient satisfaction and treatment adherence. With the advent of digital technologies in education, innovative empathy training methods such as virtual reality, simulation training systems, mobile apps, and wearable devices, have emerged for teaching empathy. However, there is a gap in the literature regarding the efficacy of these technologies in teaching empathy, the most effective types, and the primary beneficiaries -students or advanced healthcare professionals-. This study aims to address this gap through a literature review following PRISMA guidelines. A comprehensive literature search was conducted in the PsychINFO, Scopus, PubMed, and Web of Science databases using specific keywords. Inclusion criteria for articles were established, and two researchers independently rated the selected articles, resolving any disagreements by consensus. Out of 1137 articles screened, a total of 14 articles were included in this review with a total of 1285 participants, who received empathic training integrated with the use of digital technologies. Only 9 articles defined the construct of empathy, focusing on cognitive, affective, clinical, or cultural aspects. Empathy was assessed with various methods and promoted through various digital technologies, including wearables (e.g. HMDs, SymPulse™ armband) and non-wearable devices (computer monitors, Mobile Apps, Kinect System). Participants were primarily medical students (68.1%), with few healthcare workers (31.9%) and nurses (2.9%). All digital technologies effectively promoted empathy among the target population except for 3 studies that involved advanced career healthcare workers. This review highlights the potential efficacy of digital technologies in fostering empathy among medical students, though not as effectively among advanced healthcare professionals. These insights have implications for designing targeted educational programs that address the distinct needs of healthcare professionals at varying career stages. Limitations and future research directions are also discussed.

**Supplementary Information:**

The online version contains supplementary material available at 10.1007/s10916-025-02144-9.

## Introduction

### The Construct of Empathy in the Health Care Context

The physician-patient engagement is pivotal in the success of medical treatment [1, 2]. Physician-patient engagement encompasses the cooperative and interactive bond shared between the healthcare workers (HCWs) and their patients. It transcends the mere exchange of information or direction-giving by the doctor; instead, it involves a dynamic interplay where both individuals engage in decision-making, goal establishment, and treatment strategizing [3–5]. One of the key roles in establishing engagement among both parts is the physicians’ empathetic response, which refers to the ability of HCWs to deeply understand and share the feelings of their patients, creating an environment of trust and emotional support. For instance, an empathetic physician may acknowledge a patient’s fears about a diagnosis by not only providing clear explanations but also offering reassurance through active listening and compassionate communication [5]. Overall, it has been demonstrated that empathy enhanced patients’ comprehension about treatment choices and active engagement in decisions regarding therapy [6, 7].

The theoretical model of Davis [8] defines the construct of empathy into two components: cognitive, and affective. Cognitive empathy permits the physician to understand and perceive the patients’ thoughts, feelings, and perspectives without necessarily sharing those emotions [9]; whereas affective or emotional empathy implies that physicians share the emotions experienced by the patients [10]. In the area of healthcare, clinical empathy encompasses both cognitive and affective elements, but it is specifically tailored to the healthcare context [11, 12]. In particular, the concept of clinical empathy considers the ability to observe, to feel and to express the awareness of the patients’ emotions [13, 14]. For this reason, clinical empathy was defined as the heart of patient care [15]. The literature on the antecedents and consequences of empathy provides critical insights. Empathy, a multifaceted construct is influenced by individual and contextual factors and can be shaped by demographic variables like gender, age, and ethnicity [15]. Structured educational programs in healthcare and social care disciplines significantly enhance empathic skills in students and healthcare professionals, by promoting a mindset essential for effective clinical practice [12]. Early academic experiences and exposure to patient-centered care further contribute to empathy development [9].

In healthcare, empathy has profound consequences. Higher empathy levels among practitioners improve patient satisfaction and therapeutic outcomes [7]. Empathy also strengthens the therapeutic alliance, fostering trust and understanding [11]. Beyond individual interactions, Haslam [13] highlighted the broader implications of empathy in humanizing medical practice, which can lead to systemic improvements in patient care.

However, most of the investigations on physician-patient relationship do not clearly disclose the type of empathy considered or the specific component of interest. Thus, the large heterogeneity of findings is due to the lack of consensus on the dimension of empathy mostly considered in healthcare settings [16, 17]. This lack of clarity hampers the development of targeted interventions, ultimately affecting the quality of patient care and outcomes. To advance in this field, it is crucial to establish a common ground regarding the specific dimensions of empathy that are most beneficial in healthcare settings.

### Barriers to HCWs and Medical Students’ Empathy

Several barriers to empathy between HCWs and patients have been observed. One significant barrier is physicians’ anxiety and time pressure, which often prevent doctors from fully listening to patients during daily rounds. Another impediment to fostering empathy lies in the failure of many physicians to acknowledge patients’ emotional needs as integral to both illness and care. Additionally, a third obstacle surfaces when negative emotions escalate during conflicts between patients and physicians [18]. More specifically, critics have repeatedly condemned the current medical approach for evolving into a narrow and inflexible system that neglects the subjective experience of human suffering [19, 20]. Furthermore, a decrease in empathy has been observed not only among physicians in advanced careers but also among medical students [21, 22]. Among medical students, several factors contribute to this decline in empathy, including heavy academic workloads, traditional teaching methods, institutional culture, the prioritization of theoretical knowledge over humanistic aspects, burnout, and stress [23].

### Digital Empathy Training

Given the importance of empathy in clinical settings and the barriers that impede its practice, several empathy training programs have been developed for HCWs [24]. Most of the programs have been focused on asking HCWs to adopt the patient’s perspectives through imagination or, more commonly, through role-play [25]. Preliminary results have indicated positive outcomes and enhanced empathy [26]. In this context, essential support has been given by the recently adopted digital technologies as educational mediums. An example of technology adopted in education curricula is the Mobile Applications. In the context of education, and thanks to the widespread consumption of Mobile Apps, many higher education organizations have implemented mobile learning to offer flexibility in learning [27, 28]. For example, a Mobile App was used to provide emergency care for infant airway obstruction and the instructional content consisted of causes, frequency, suspicious signs, and emergency care [29].

Another digital technology that has exploded in educational context is Virtual Reality (VR). VR refers to a computer-generated environment that simulates a realistic sensory experience, often including sight, sound, and sometimes touch. Users typically experience VR through a headset or goggles that immerse them in a simulated environment, allowing them to interact with and navigate through digital spaces or scenarios as if they were physically present. In medical education, it has been adopted to train HCWs in surgical practice, as it allows for the simulation of a realistic medical experience [30, 31]. This technique has been demonstrated to be efficacious in fostering empathy in people belonging to outgroups, including gender, age or ethnicity [32–34]. For educational purpose the virtual avatar has also been adopted. It is a digital representation of a user or individual within a virtual environment projected through a computer screen or Head Mounted Display. Avatars can be customized to reflect various characteristics, including appearance, clothing, accessories, and sometimes personality traits. Within medical training, researchers have used virtual patients, a subset of virtual agents, to support both the acquisition of theoretical knowledge as well as communication skills [35, 36]. The published literature demonstrates that utilizing virtual patients, whether as independent learning modules or alongside traditional classroom teaching, enhances students’ proficiency in clinical reasoning, ethical decision-making, and communication skills [37].

In conclusion, digital technologies seems to represent valuable tools in supporting empathy training through specific features. These include flexibility in learning, the ability to simulate realistic medical experiences, and the option to customize virtual avatars or environments. By leveraging these capabilities, digital technologies can provide structured and impactful training opportunities. However, considering the growing use of technologies in education including Mobile Apps and Virtual Reality and Virtual Patients, few literatures investigate its potentiality in empathy training.

### Objectives

This systematic review aims to address a critical gap in the literature by summarizing the most recent (last 10 years) research on the role of digital technologies in fostering empathy among healthcare workers and medical students. Specifically, the objectives of this review are: (1) to evaluate the benefits and the effectiveness of digital technologies in enhancing empathy within healthcare contexts; (2) to identify existing research gaps and propose areas for future investigation; and (3) to provide actionable insights for stakeholders, including educators, policymakers, and practitioners, regarding the integration of digital empathy training into healthcare education. By doing so, this review seeks to contribute to both the academic understanding of digital empathy and the practical advancement of empathy-based training in the healthcare field.

### The Present Study

The research questions of the present review (RQ) are:

#### RQ1

How is the construct of empathy defined and measured by the selected literature?

#### RQ2

On which factors (i.e., affective/cognitive) of empathy do the various digital empathy training primarily focus?

#### RQ3

Could digital technologies be effective to promote and foster empathy among HCWs? If yes, which empathy factors are more propense to change?

**RQ4** Which type of digital technologies are more effective?

#### RQ5

Who benefits more from empathy training, medical students or HCWs in advanced career?

## Method

**Fig. 1:**
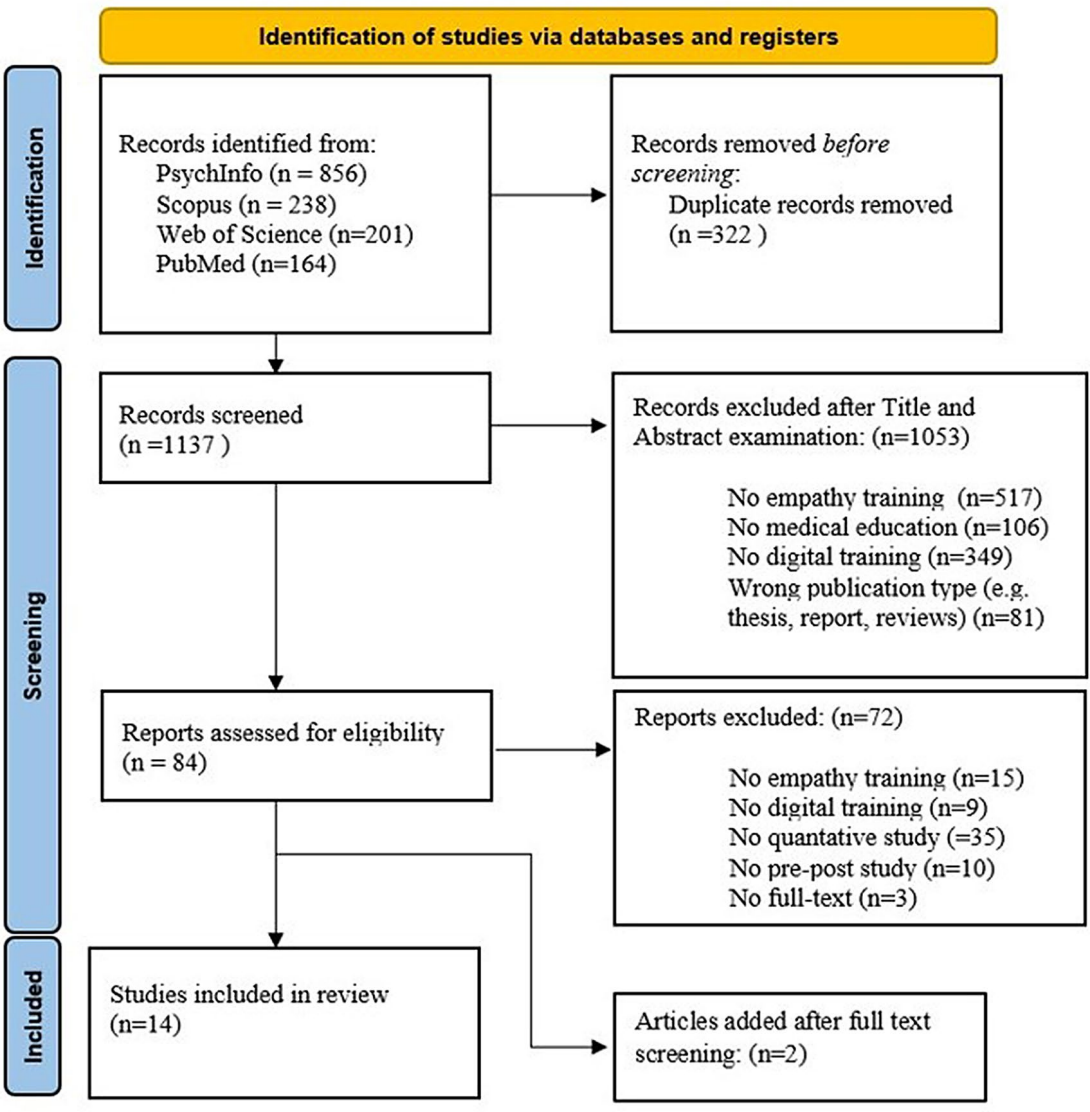
Flowchart depicting the identification and selection of the articles

A systematic review was conducted to extract recently published scientific papers that focused on using digital technologies to induce empathy in a population of medical students or HCWs. The identification, screening, and selection process is summarized in Fig. 1. The review focused on the last 10 years (from 2014 onward) since in this time frame digital technologies have known rapid development with dramatic changes in hardware, software, and cost feasibility and their application in social and clinical fields emerged. This review adheres to the Preferred reporting items for systematic reviews and meta-analyses (PRISMA) guidelines ​ [38]. A prisma statetemet is uploaded as appendix.

### Eligibility Criteria

To be included in this review, the studies should involve: (1) a population of HCWs or medical students, (2) examine the change in empathy (3) the use of digital technologies, and (4) be quantitative study. Studies were excluded if they (1) did not provide an assessment of empathy, (2) empathy was assessed only after training and not at baseline; (3) were not experimental studies (e.g., systematic reviews, protocols, or book chapters); and (4) were not written in English language.

### Search Strategy

A systematic literature search was conducted on the following electronic databases: Ebescohost (in particular PsycINFO), PubMed, Web of Science, and Scopus. All database searches were performed in March 2024 by entering the following keywords and Boolean operators in the title and abstract section: *(((empathy OR compassion OR “interpersonal relationship”) AND (training)) AND (medical OR medicine OR health OR healthcare)) AND (digital OR virtual OR technology).* The search results were then filtered for year (only the last ten years) and language (only English language). Additional papers were identified from the citations from the retrieved references.

### Quality Assessment of the Articles

To assess the quality of the selected studies we employed the Standard Quality Assessment Criteria for Evaluating Primary Research Papers from a Variety of Fields (QUALSYST) [39]. For the present review only the quantitative scoring system was employed by 2 authors for all the included articles.

## Results

### Search Outcome

The initial screening identified 1459 articles. Most of them were extracted from the EBSCOhost (PsychINFO) (856/1459, 58.7%) and Scopus (238/1459, 16.3%) databases. The other articles were extracted from Web of Science (201/1459, 13.8%) and, PubMed (164/1459, 11.2%). After removing duplicates, 322 publications were identified and individually assessed based on the study title and the information provided in the abstract (Fig. 1). Out of the total 1137 papers, 1053 (92.6%) were disqualified based on the specified inclusion and exclusion criteria. The full texts of 84 (7.4%) articles were reviewed to determine their eligibility for inclusion. Of these 84 studies, 15 (17.8%) did not include specific training on empathy, 9 (10.7%) included empathy training but did not involve the use of digital technologies, 35 (41.7%) were not quantitative studies, 10 (11.9%) did not compare empathy levels before and after the training, and it was not possible to obtain the full text of 3 (3.6%) papers; therefore, they were excluded. Finally, 2 (2.4%) papers were added based on the reference found during the full-text screening process. A total of 14 (16.7%) articles met all the inclusion criteria and were included in this review with a total of 1285 participants, who received empathic training integrated with the use of digital technologies.

### Empathy Construct Definition and Assessment (RQ1)

Of the 14 articles included, only 9 (64.2%) defined what the authors considered empathy, whereas the remaining 5 articles [40–44] did not clearly define the construct (see Table 1). Of the articles that defined the concept of empathy, 4 (28.6%) focused on cognitive and affective factors of empathy [45–48], whereas 4 (28.6%) focused more deeply on the concept of clinical empathy [49–52], and 1 (7%) focused on cultural empathy [53].

**Table 1:** Empathy definitions

Reference	Empathy definitions	Empathy Factors
Anishchuk et al., 2023 [45]	Clinical empathy is not simply “detached concern” but rather emotional atonement, and it describes the clinical skill of emotional resonance and curiosity about the meaning of a clinical situation for the patient. It is a clinical skill involving the active assessment of a patient’s emotions and responding to patient cues. Most recently proposed as “empathic concern,” clinical empathy can be understood as “the attitude of genuine interest towards the experience of the other” which comes from an “engaged curiosity”.	Clinical Empathy
Olsen et al., 2020 [47]	A two-phase process: (a) understand and appreciate another person’s feelings and emotions and (b) communicate understanding back to the patient in a supportive way with a focus on the communication through voice.	
Foster et al., 2016 [46]	Empathy is a complex phenomenon, conceptualized as having an affective component (the ability to share emotional experiences), a cognitive component (understanding the emotions of another person), and a behavioral component (the clinician’s verbal and nonverbal expression of empathy toward the patient).	
Palanica et al., 2018 [48]	The ability to understand and accurately acknowledge the feelings of another, eliciting a more receptive response from the observer. Clinical empathy involves both cognitive and affective components, which include (1) understanding the patient’s situation, thoughts, and feelings, (2) verifying its precision with the patient, and (3) responding to the patient in a helpful manner.	
Halton et al., 2018 [50]	Empathy can be described as having two interrelated dimensions: cognitive and affective. Cognitive empathy measures the skills-based aspect of learning, where a person is able to recognize and understand another’s experience (Kourakos et al., 2018). Then affective empathy links to the transformative aspect of the learning cycle, where the understanding resonates emotionally with the individual and they start to be able to interpret their knowledge, exploring concepts beyond the facts they are presented with (Kourakos et al., 2018).	Cognitive and Affective Empathy
McCalla et al., 2023 [51]	Empathy encompasses knowing, comprehending, and perceiving what another person is experiencing,	
Gilbert et al., 2023 [49]	Empathy is a complex and dynamic task that requires a sustained cognitive effort and emotional maturity.	
Yao et al. 2022 [52]	To have empathy is to understand the value and scope of another’s felt emotion and consider this affective state from their perspective	
Everson et al., 2015 [53]	Cultural empathy refers to the learned ability to perceive and share experiences through the unique lens of values, beliefs and perspectives of people from cultural backgrounds different to one’s own.	Cultural Empathy
Hess et al., 2022 [40]	No clear definition is provided	
Manuel et al., 2023 [41]	No clear definition is provided	
Quail et al., 2016 [42]	No clear definition is provided	
Sauve et al., 2022 [43]	No clear definition is provided	
Tong et al., 2017 [44]	No clear definition is provided	

To respond to RQ1, we also evaluated how the included studies assessed the empathy change in the participants. Most of the studies used the self-report Jefferson scale for Empathy [54] (4/14, 28.6%) and the expert-rated scale Empathic Communication Coding System [55] (ECCS) (3/14, 21.4%). Of the remaining papers, each study utilized a different scale to measure empathy or related constructs^56^–^58^ . For details on these scales, please refer to Table 2. In conclusion, among the included studies, the concept of empathy was scarcely defined and assessed with great heterogeneity.

**Table:** Table 2 Summary of the included studies

Study	Sample	Study design	Empathy Measures	Type of Technology	Outcomes	Quality Assessment	Training duration	Perspective	Interactivity
Anishchuk et al., 2023 [45]	37(F = 28), Dental nurse (*N* = 3), Dental hygiene (*N* = 7), Dental science (*N* = 27)	Single group pre-post training assessment.	Jefferson Scale of Empathy	Computer (video presentation + online lessons)	Significant increase between pre-post training (t (36) = 3.6 *p* = 0.001) and effect size d = 0.59	0.82	3 weeks	Third person/Non wear/Dr.&Pz.	No
Olsen et al., 2020 [47]	42(F = 37), Nursing student	Within pre-post design, VP condition, Role Play condition	Reflect on empathy displayed on 1–5 likert	Computer-based virtual patients	Significant increase between pre-post, t(157) = 5.15, *p* < 0.001. Significant impact of the condition, t(38) = 3.13, *p* < 0.01, with empathy rating higher in the role-playing condition.	0.46	1 session per condition	First person/Non wear/Dr.	Yes
Foster et al., 2016 [46]	70 (F = 33), Medical student	RCT, CG = Control VP, EG_2_ = Backstory VP, EG_1_ = Empathy feedback VP	Empathetic Communication Coding System	Computer-based virtual patients	Only the difference between the empathy-feedback VP [mean (SD), 2.91 (0.16)] and the backstory VP [mean (SD), 2.20 (0.22)] groups was statistically significant. (*P* = 0.0277).	1.00	1 session per group + 1 SP interaction	First person/Non wear/Dr.	Yes
Halton et al., 2018 [50]	104(F = 66), Employees of pharmaceutical company	Mixed methods pre-post design	Toronto Empathy Questionnaire	Mobile APP	Significant increase between pre-post training (t (73) = 3.1, *p* = 0.002) and effect size d = 0.45	0.55	36 h constructed narratives	First person/non wear/Pz.	Yes
Hess et al., 2022 [40]	35 (F = 33), Home health professional	Single group pre-post training assessment.	Interpersonal Reactivity Index	Web symposium (computer)	No significant change in empathy	0.55	3 10–12 min session of VR livestreamed to the participant	First person/Non wear/Pz.	No
Manuel et al., 2023 [41]	72(F = 68) Physician(*N* = 57), Nurse(*N* = 31) Trainee(*N* = 19).	RCT, EG = Virtual Reality, CG= asynchronous web-based platform.	Motivational Interviewing Treatment Integrity behavioural coding system.	Computer-based and web-based training	Significant difference from before to after the empathy training for the EG (*p* = 0.04) compared to the CG (*p* = 0.53).	0.78	90-minute training sessions 2 weeks apart	Third person/Non wear/Pz. & Dr.	No
McCalla et al., 2023 [51]	69(F = 57). Psychology and Education (*n* = 17), Healthcare (*n* = 43), Medicine (*n* = 7).	Single group pre-post training assessment.	Jefferson Scale of Empathy Health Care Provider Students Version.	Virtual Reality (Pico G2 4 K head-mounted displays).	Significant difference from before to after the empathy training (p = < 0.001).	0.61	1 session	Third person/wear/Pz.	No
Palanica et al., 2018 [48]	27 (F = 10) helathcare professionals (EG = 13; GC = 14).	RCT: EG = perform motor function tasks (e.g., buttoning a shirt and printing out one’s name) with SymPulse™, CG = perform motor function tasks without SymPulse™.	Jefferson Scale of Empathy Health Care Provider Students Version (only pre-test, trait empathy); State Empathy Scale (only post-test, state empathy).	SymPulse™ is an arm band that simulate the involuntary muscle tremors of Parkinson patients.	Trait empathy = no significant difference between condition (*p* > 0.90). State empathy = significant difference between conditions (*p* < 0.01).	0.75	1 session	First-person/wear/Pz.	No
Quail et al., 2016 [42]	62 (F = 62) undergraduate speech pathology students.	RCT: Nursing Home (G1), Standardized Patient (G2), Virtual Learning (G3).	Jefferson Scale of Empathy Health Care Provider Students Version.	Computer based Virtual Patients	Significant difference between the pre and post-test only for the Nursing Home (CG) condition (*p* < 0.01).	0.78	5 weeks, 1-hour por week	First person/Non wear/Dr.	Yes
Sauve et al., 2022 [43]	29 (F = NA) physician students-	Single group pre-post training assessment.	Ethnocultural Empathy Scale.	Mobile App: Stand Up for Indigenous Health (SU4IH).	Significant difference on empathy score between pre and post test (*p* < 0.001).	0.57	1 session	First person/Non wear/Pz.	Yes
Yao et al., 2022 [52]	25 (G1) and 27 (G2) (F = 25) healthcare professionals.	Two non-randomized conditions: Post-interview Feedback Condition (G1), Scaffolded Ping-pong Feedback Condition (G2).	Empathic Communication and Coding System.	Computer based Virtual Patients	No significant difference between groups for high-empathy level responses (*p* = 0.58), medium (*p* > 0.05), and low-empathy (*p*= 0.06).	0.67	3 weeks (2nd week in unactive)	First person/non wear/Dr.	Yes
Tong et al., 2017 [44]	15(F = 4), Healthcare job related (*N* = 2), Not clearly defined (*N* = 13)	Mixed method pre-post training assesment	Revised Compassion for Others Scale and willingness of help (1 item) (Pommier, 2011)	Body motion sensors	No significant difference in level of empathy. Significant difference in willingness to help t (13) = 2.132, *p* = 0.026 and effect size d = 0.50	0.55	1 session	Third person/Non wear/Pz.	Yes
Gilbert et al., 2023 [49]	72(F = 48)								
Medical student	One group longitudinal design	Empathic Communication Coding System	Computer-based virtual patients	There was a statistical difference in ECCS scores based on empathic opportunity (χ2 [3] = 7.66, *P* < 0.05) but not based on the order of interview series over time (χ2 [5] = 5.51, *P* = 0.36).	0.65	4 VP interviews during 12 weeks courses	First person/Non wear/Dr.	Yes	
Everson et al., 2015 [53]	460(F = 405), Nursing student	Single group pre-post training assessment.	Modified version of the Kiersma Chen, Empathy Scale (Kiersma et al., 2013).	Head mounted System	On average, participants reported significantly higher mean scores on the MKCES post-simulation 49 24 (SD = 518) compared to pre-simulation 47 86 (SD = 464); t (459) = 4639, *p* < 0001.	0.77	1 session	First-person/wear/Pz.	No

### Aspects of Empathy Addressed by Training Programs and Their Theoretical Backgrounds (RQ2)

Empathy training programs in healthcare employ a variety of approaches, each targeting different facets of empathy, including emotional resonance, cognitive perspective-taking, and practical clinical application. A significant portion of these programs is devoted to refining *interpersonal communication skills*, with a particular emphasis on fostering clinical empathy. For example, Manuel and colleagues [41] underscored the importance of techniques like motivational interviewing in enhancing communication between HCWs and patients. Similarly, Olsen and colleagues [47] and Gilbert and colleagues [49] utilized virtual patients to improve communication skills within clinical scenarios. Feedback mechanisms have also been highlighted as crucial for enhancing empathetic performance, as evidenced by the work of Foster and colleagues [46] and Yao and colleagues [52] who demonstrated the efficacy of real-time feedback in enhancing verbal empathy. For a review on conversational agent and patients’ engagement [59]. Another significant focus of empathy training programs is on the *cognitive aspect* of empathy. Some of the training incorporates exercises and simulations that encourage HCWs to understand and appreciate the diverse cultural backgrounds and experiences of their patients. For instance, Everson and colleagues [53] utilized immersive cultural simulations to expose nursing students to scenarios in developing country hospital wards, thereby enhancing their cultural empathy. Similarly, Sauve and colleagues [43] developed simulations aimed at promoting intercultural empathy, such as the Stand Up for Indigenous Health (SU4IH) program.

In some instances, training programs adopted a comprehensive approach, addressing *cognitive and emotional dimensions of empathy*. Anishchuk and colleagues [45], for instance, developed a module on clinical empathy that focused on recognizing and understanding patients’ feelings and experiences, as well as effectively communicating this understanding to patients. Likewise, Hess and colleagues [40] proposed interventions for Parkinson’s disease that encompassed lectures, breakout sessions, and immersive virtual reality experiences, aimed at enhancing empathetic interactions with patients. Beyond cognitive and emotional empathy, certain programs explore alternative dimensions of empathy. For example, Palanica and colleagues [48] introduced the construct of *tele-empathy*; a class of technology used to accurately identify, digitize, and characterize symptoms in a specific patient, generating a representative physiological response in a non-patient to elicit empathy for a particular health condition. Tong and colleagues [44] investigated the potential of embodied video games to raise awareness and foster positive attitudes among HCWs, although the impact on empathy was less pronounced.

In conclusion, the majority of articles included referred to clinical empathy, with a specific focus on communication skills and understanding of different cultural backgrounds of patients or the implications of their symptoms.

### Type of Technology Used (RQ3) and Effectiveness (RQ4)

Main results are synthesized in Table 2. We considered the different types of technologies employed by the articles included and their efficacy in promoting empathy. All the studies documented improvements in some dimension of empathy among the target population, except for 3 studies (21.4%) [40, 44, 52]. Two of the studies that did not show significant improvements were computer-based training that used Virtual Patients [40, 52] and one of them used body motion sensors [44]. In fact, the digital technologies adopted by the investigators can be divided into *wearable or no-wearable*. The former are: the Head Mounted Display (HMD) adopted in 2 [51, 53], out of 14 studies (14.3%) and the wearable arm band SymPulse™ used in 1 [48] study (7.1%). The no-wearable technologies are the computer monitor, used in 8 [40–42, 45–47, 49, 52] out of 14 studies (57.1%), the Mobile Apps, used in 2 [43, 50] out of 14 studies (14.3%), and the Kinect System [52] (1/14, 7.1%). Thus, the majority of the interventions (10/14, 71.4%) used no-wearable technology including computer and Mobile Apps, and only 4 (28.6%) of them used wearable devices including HMD and sensors.

The studies where Mobile Apps were applied [43, 50] and those that used VR- the Head Mounted Display (HMD) [51, 53] showed significant improvements of empathy after training. Palanica and colleagues [48] used the wearable armband SymPulse™, a device to deliver electrical stimulation aimed at mimicking the Parkinson’s tremors experienced by the patients and showed promising results in promoting emotional empathy in the experimental group compared to control. Similarly, Tong and colleagues [44] showed no significant improvements in the level of empathy but a significant improvement in willingness to help using a body motion sensor to simulate the experience of a chronic pain condition. However, none of the studies analysed the individual components of empathy, such as emotional and cognitive empathy. Instead, the authors reported the results for the overall construct.

Considering the *point of view of the digital experience*, most of the studies (10 out of 14, 71.4%) employed a first-person view, where the user is the protagonist of the scenario, and 4 studies (28.6%) used a third-person view, where the user observe a virtual scenario. Regardless of the point of view, 5 of the included studies were implemented from the doctor’s perspective, 3 included both the perspective of the doctor and the patient, and 7 (50%) of the studies were implemented from the patient’s perspective. The majority of investigations documented some significant improvements in empathy levels from pre- to post-intervention except for one study using the third-person perspective [44], which employed the use of a no-wearable device from the patient’s perspective, and two first-person studies [40, 52], which employed both no-wearable technologies from the patient or doctor perspectives.

Finally, the digital technologies applied in the 14 included articles of this review differed in terms of *interactivity*. Most of the empathy training included the possibility of interacting with the digital environment (8/14, 57.2%). Five of them were text-based answers or questions to an avatar (35.7%), and 2 were based on Mobile App interactions (14.3%), or interaction with the avatar (1/8, 7.2%). Conversely, 6 studies applied a no-interactive digital training: 5 of them (42.8%) were based on the passive view of 360-degree video regarding the patient’s condition or web educational material. 1 study showed a different approach from all the other interventions, by using a device able to record the muscle activity of a patient with Parkinson’s disease to give the experience to live with muscles impairments and tremors.

Of the 3 studies that showed no significant changes on empathy scores, two were interactable [44, 52] and one was not [40].

### Targeted Population (RQ5)

The screened articles included a population mainly composed of medical students (780/1146, 68.1%), and HCWs. (366/1146 31.9%). Considering the category of the HCWs, nurses were the less represented group with only 34 (2.9%) participants included in the studies. All of the studies that did not show a significant improvement in empathy levels [40, 44, 52] included only physicians or healthcare professionals.

### Risk of Bias Assessment

The mean quality score for 14 articles was 61% ranging from 46% [47] to 100% [46]. The main reasons for lower scores were the lack of blinding of investigators or participants (71.4%), and the lack of a control group (57%). The higher scores included appropriate study design to respond to research questions and described and presented appropriate analysis. The detailed quality assessment of each study is provided in Table 2.

## Discussion

For a successful clinical relationship, empathy expressed by medical students and HCWs in advanced career, plays a key role in cognitively and affectively enhance the understanding of patients’ emotions [12]. However, the increasing rates of burnout, excessive workload and emotional distress among HCWs are seriously compromising the capacity to build a positive and collaborative physician-patient relationship [18]. For this reason, several training programs were added to medicine educational curricula supported by digital technologies [43, 49, 50]. However, to the best of our knowledge, no conclusive data are available for explaining if these digital technologies could be effective in training and fostering empathy and, if yes, which types of technologies were more effective and what targeted population would benefit the most from them (medical students or HCWs in advanced careers). The present review aimed at filling this gap and investigating if digital technologies could be the potential medium to foster empathy in healthcare professionals, both students and HCWs.

In regards to RQ1 and RQ2 the first important observation that emerged from the review is that there is not a clear consensus about the definition of empathy construct. As we can see from Table 1, the authors used several empathy definitions including clinical empathy, cognitive and affective empathy, and cultural empathy. However, several studies did not define the construct, but focused primarily on the technological aspect of the research, without a clear theoretical background (RQ1). To effectively develop empathy training, future research should adopt a theory-driven approach, should clearly define the specific aspects of empathy to be targeted, should outline the methods to address them, and justify the reasons for these choices. This lack of consensus is also reflected in the different empathy measures adopted by the authors of the included studies to assess the change from baseline to after the empathy training. In fact, out of the 14 included articles, 9 different empathy questionnaires were assessed. The lack of a clear definition of the construct in these studies, coupled with a predominant focus on the technological aspects of the research, likely contributes to the heterogeneity of the results presented in the present review (RQ2).

In regard to RQ3 and RQ4, two types of digital technologies used in the included studies emerged: wearable and no-wearable ones. The former are the VR-HMD and the armband SymPulse™. The latter are the Mobiles Apps, the computer-based training and the motion sensor Kinect. Those technologies differed also in terms of interactivity and perspectives (doctors’ point of view vs. patients’ point of view). Therefore, they could provide participants with different types of experience in terms of presence and immersion in the clinical scenario, that could influence the effectiveness of the empathy training and the final outcomes of the studies (RQ3). All the digital technologies adopted in the included studies effectively promoted some dimensions of empathy among the target population except for 3 studies. Two of the studies that did not show significant improvement were computer-based training (no-wearable, less immersive) that used Virtual Patients [40, 52] and one of them used body motion sensors [44] (RQ4).

All the Mobile App studies and those that included HMD showed efficacy in improving empathy after the training. The interactive and immersive nature of these technologies tends to engage users more deeply, fostering a stronger empathetic response [60] compared to traditional teaching. For example, in the study of Halton and colleagues [50] the participant takes the perspective of the patient with inflammatory bowel disease and is prompted to face some of the challenges that are typical of this disease to experience barrier and limitation that the illness poses on the patients. Also, the VR-HMD permits to experience a high sense of presence with the content and a high sense of embodiment [61] with the virtual patients or HCWs. For example, in the study of McCalla and colleagues [51] a participant is invited to take the third-person perspective of an older woman with type 2 diabetes and results indicated a high degree of identification with the avatar.

One interesting result comes from the wearable armband SymPulse™ used by Palanica and colleagues [48] which showed promising results in promoting empathy. This study used a device to deliver electrical stimulation aimed at mimicking the Parkinson’s tremors experienced by the patients.

Regarding RQ5, all training programs that failed to significantly improve empathy involved experienced HCWs, regardless of the differences in interactivity and engagement among digital devices. This lack of efficacy could be due to the fact that HCWs in advanced careers need different kinds of training compared to medical students, and that the empathy skills may change over time during the medical career [21, 54, 62]. Alternatively, medical students are often younger and, as digital natives, they could have more familiarity and acceptability of the digital training as compared to older HCWs who could be more sceptical about these innovations in medical training [63].

In conclusion, the findings of this review seem to suggest that there is no “one size fits all” solution in the context of empathy training. Different technologies offer different experiences of presence and immersion, which can influence the training outcomes. Therefore, future training programs should carefully consider the choice of technology and aim for a tailored approach to meet the diverse needs of healthcare professionals. For example, Ma and colleagues [64] demonstrated that playing a game about a child diagnosed with cancer in VR resulted in a greater sense of spatial presence and increased empathy compared to non-VR on a nursing students’ population. Additionally, taking the perspective of the virtual healthcare provider, compared to the patient’s family perspective, elicited higher levels of empathy in VR compared to 2D computer [64]. These pilot investigations seem to suggest that the stronger immersion generated by the VR technologies may lead to a greater change in empathy scores, and the point of view adopted by these technologies may act as a moderator.

Additionally, Foster and colleagues [46] reported greater empathy levels only when virtual patients provided feedback on participants’ empathic responses. Thus, a greater emphasis should be placed on enhancing empathic communication skills through these trainings, whether using virtual patients or other methods, to ensure a comprehensive development of both cognitive and affective empathy in healthcare providers. Nonetheless, most of the studies that employed virtual patients did not provide any kind of feedback on the response offered by the participant.

## Conclusion

This review aimed to address the effectiveness of digital technologies in fostering empathy among healthcare professionals. A key observation is the lack of consensus on the definition of empathy, leading to varied interpretations and inconsistent results in empathy training programs (RQ1). To effectively develop empathy training, future research should adopt a theory-driven approach, clearly define the specific aspects of empathy to be targeted, outline the methods to address them, and justify the reasons for these choices (RQ2).

The review identified two types of digital technologies: wearable (e.g., VR-HMD and SymPulse™ armband) and non-wearable (e.g., mobile apps and motion sensors), all effective in improving empathy (RQ3). However, effectiveness varied between medical students and experienced HCWs, suggesting the need for different training approaches for different target populations (RQ4).

Overall, the findings indicate that there is no universal solution for empathy training, and future programs should carefully select technology and tailor approaches to meet the diverse needs of healthcare professionals.

## Limitations and Future Directions

The review presents some limitations. First, the great heterogeneity in the assessment of empathy made the comparisons among the different interventions difficult at this stage of the research. It is important for future research to unify the use of a standard questionnaire to measure the core characteristics of empathy in the healthcare context. Finally, another limitation is that none of the included studies examined the mechanisms underlying the effectiveness of the empathy training through careful experimental manipulation.

Future studies should increase the sample size, by including a variety of HCWs, with different professional seniority and should adopt a randomized controlled design. Furthermore, future studies should include measurements that help to understand empathy not only in terms of psychometrics, but also in terms of actual behavioural change. For example, instead of solely relying on self-reported empathy scales, researchers could observe and evaluate changes in participants’ real-world behaviours (e.g., increased in prosocial actions).

## Electronic Supplementary Material

Below is the link to the electronic supplementary material.

Supplementary Material 1

## Data Availability

No datasets were generated or analysed during the current study.
